# Fulminant Pseudomembranous Colitis Presenting as Sigmoid Stricture and Severe Polyposis with Clinical Response to Intracolonic Vancomycin

**DOI:** 10.1155/2016/4609824

**Published:** 2016-02-29

**Authors:** Sai Wah Cheung, Kin Kong Li

**Affiliations:** Division of Gastroenterology and Hepatology, Department of Medicine and Geriatrics, Tuen Mun Hospital, Tsing Chung Koon Road, Tuen Mun, New Territories, Hong Kong

## Abstract

*Clostridium difficile* infection (CDI) is the most common cause of antibiotic-associated diarrhea. Severe diseases carry significant morbidities such as septic shock, acute kidney injury, bowel perforation, and mortality. Immunocompromising conditions increase the risk of developing the disease but whether these individuals suffer a more fulminant course or warrant a more potent first-line treatment is still controversial issue. Hereby we report a case of a cirrhotic patient with life-threatening pseudomembranous colitis complicated by colonic stricture, initially refractory to standard treatment but with subsequent improvement on intracolonic vancomycin.

## 1. Introduction

*Clostridium difficile *(*C. difficile*) is an anaerobic Gram-positive spore-forming organism which was first identified to be the cause of antibiotic-associated diarrheal disease and pseudomembranous colitis in 1978 [1, 2]. Oral vancomycin was approved by the US Food and Drug Administration (FDA) in the 1980s as the first proven treatment for* C. difficile* infection (CDI) and metronidazole was subsequently found to be equally effective [2, 3]. The incidence of CDI appeared stable since its discovery until the 20th century, when it became a principal nosocomial infection with increasing incidence, severity, rate of recurrence, mortality, and more refractoriness to treatment [4–8]. CDI is the most common cause of antibiotic-associated diarrhea. Severe disease is associated with significant morbidities such as septic shock, acute kidney injury, ileus, bowel perforation, and, ultimately, increased mortality. Immunocompromising conditions including malignancy, receipt of chemotherapy and/or corticosteroid therapy, organ transplantation, and cirrhosis increase the risk of developing the disease. However, whether these individuals suffer a more fulminant course or warrant a more potent first-line treatment is still controversial issue [9, 10].

## 2. Case Presentation

A 59-year-old gentleman with no significant past medical history was admitted to the hospital for acute onset of profuse, watery diarrhea with more than ten bowel movements a day. He had a dental extraction one month priorly and received oral amoxicillin/clavulanate for three days.

Upon admission, he was febrile and severely dehydrated and had evidence of shock. The physical examination revealed mild distension and diffused tenderness over the abdomen associated with mild ankle edema. Plain X-ray of the abdomen showed prominent small bowel and large bowel loop up to 5 cm in diameter. There was mild leukocytosis with WBC of 11.58 × 10^9^/L, and there were severe hyponatremia and hypoalbuminemia with Na level 118 mmol/L and albumin level 24 g/L; the serum creatinine was 72 *μ*mol/L and the urea was 11.9 mmol/L. Liver biochemistry was normal. The initial resuscitation included aggressive fluid replacement, electrolyte supplementation, and initiation of empiric oral antibiotic therapy with ciprofloxacin.

On day 3, the blood culture grew group D* Salmonella* species and stool* Clostridium difficile* PCR was positive. Otherwise, the stool was negative for bacterial culture, ova, and cyst. As the* Salmonella* bacteremia was considered the culprit for the septic shock and the CDI was thought to be a less major problem at that junction, antibiotics were switched to oral metronidazole for 7 days to treat CDI and intravenous ceftriaxone to treat* Salmonella* bacteremia accordingly. Despite the medical treatment, his diarrhea persisted and was complicated by voluminous perrectal bleeding resulting in anemia requiring supportive transfusion.

**Figure 1:**
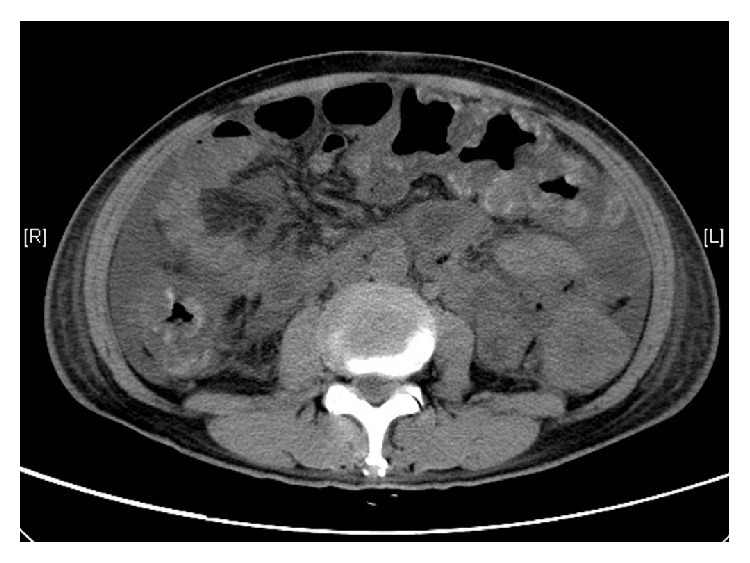
Abdominal computer tomography shows diffuse irregular mural thickening of the large bowel loops with scattered mucosal and submucosal hyperdensity suggestive of hemorrhage.

Computer tomography of the abdomen showed cirrhosis of liver with gross ascites and a segment V/VI 3 cm hepatocellular carcinoma. Colonic findings were diffuse irregular mural thickening and submucosal edema with scattered mucosal and submucosal hemorrhage along large bowel loops from the rectum to ascending colon (Figure 1).

**Figure 2:**
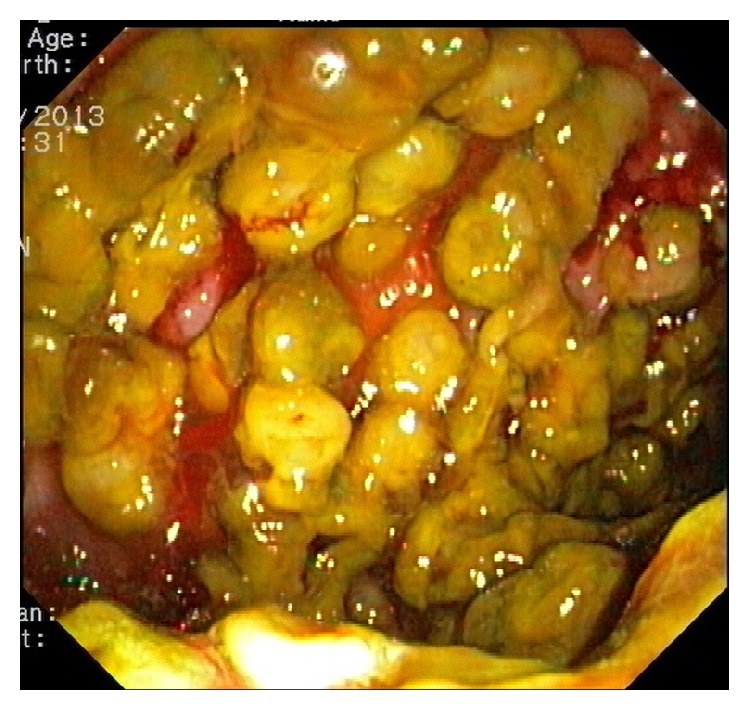
Endoscopic view of the sigmoid colon shows multiple polypoid lesions covered with a thick yellowish cap and active mucosal oozing, sparing small areas of normal mucosa.

Colonoscopy showed severe proctitis with small roundish yellowish plaques in the rectum. The sigmoid colon was macroscopically covered with multiple polypoid lesions having a thick yellowish cap, with small areas of normal mucosa spared. Active mucosal oozing along the inflamed region was noted (Figure 2). There was an almost complete stricture of the mid sigmoid colon caused by the ingrowth of the polypoid lesions hindering further advancement of the colonoscope. Multiple colonic biopsies confirmed the histological diagnosis of pseudomembranous colitis with inflamed fibrinous exudate admixed with necrotic debris and Kayexalate crystals; there was no evidence of adenoma, dysplasia, or malignancy.

The patient's HbsAg was positive with a HBV DNA serum level of 1.21 × 10^6^ IU/mL, for which entecavir was initiated. The patient was managed as fulminant pseudomembranous colitis and treated with intravenous metronidazole and oral vancomycin according to the most updated IDSA guidelines. However, the diarrhea and perrectal bleeding were refractory to the combination treatment after 5 days and rectal vancomycin enema 500 mg in 100 mL NS every 6 hours was added as an adjunct. The diarrhea and dysentery improved within 2 days of the initiation of rectal vancomycin and the symptoms resolved completely after 5 days of rectal enema. The rectal vancomycin was discontinued after 7 days and intravenous metronidazole and oral vancomycin were given for 14 days in total. On discharge, his creatinine level was 38 *μ*mol/L and the urea and electrolytes were normalized.

The patient remained asymptomatic and repeat colonoscopy 3 weeks after resolution of symptoms identified only mild diffuse erythematous colonic mucosa with no pseudomembrane appearance. There were no strictures or resistance with scope advancement to the terminal ileum.

## 3. Discussion

CDI was first identified in 1978 and its incidence, recurrence, and disease severity have been reported to be increasingly virulent [4, 11]. Clinical manifestation mimics the symptoms of other causes of gastroenteritis and includes diarrhea, abdominal pain, fever, leukocytosis, and rarely ileus or perrectal bleeding. The clinical spectrum varies from asymptomatic carrier or mild self-limiting disease to severe fulminant colitis which may lead to bowel perforation, warranting an urgent colectomy. The diagnosis should be suspected early in any patients with diarrhea, particularly those with immunocompromised status. CDI can be confirmed by stool tests for bacterial culture, toxigenic culture, toxic EIA tests, or PCR assays and colonoscopy could be offered if urgent endoscopic diagnosis is required [10, 12].

Patients with cirrhosis and CDI had been shown to carry a higher mortality, length of stay, and hospitalization charges compared to patients with CDI alone after controlling for age and comorbidities [9]. This phenomenon could be pathophysiologically attributed to the impaired local gut immune response, increased bowel wall edema, and poor intestinal motility. Additionally, the cirrhotic patients are prone to the use of antibiotics for the treatment of infection or prophylaxis of spontaneous bacterial peritonitis [13]. Also, proton-pump inhibitors are used frequently in cirrhotic population for the prevention of postbanding esophageal ulcers, where its use per se is associated with increased risk of CDI [14, 15]. Notably, a low serum albumin level, which is universally associated with advanced stage of cirrhosis, has been well reported to be a predictor of severe or recurrent CDI [14, 16, 17]. All these factors could lead to increased severity of CDI in cirrhotic patients and our case highlights an extreme presentation of fulminant colitis with massive perrectal bleeding, sigmoid obstruction, and pseudopolyposis of the involved colonic segment.

Pseudopolyposis appearance of the colon is a rare endoscopic finding in pseudomembranous colitis [18, 19]. In other cases, CDI was reported to have exclusive localization over true adenomatous polyps with sparing of the normal colonic mucosa [18]. These presentations are extremely unusual and have been only reported in a few case reports. The exact pathophysiology for this striking mucosal change is unknown but it was proposed that dysplastic changes of the colonic epithelium might represent a site of greater affinity for* C. difficile* toxin or tropism of* C. difficile* bacteria with the consequent formation of pseudomembranes [18]. Structural disease due to stricture was also infrequently reported in the literature in both adult and pediatric patients. It usually accompanied severe colitis with high fever, leukocytosis, perrectal bleeding, abdominal pain, and distension with or without signs of ileus. Surgical intervention with colectomy as the salvage therapy seems to be invariable in the scanty reports [20, 21] and our trial of rectal vancomycin had been used successfully to act as an adjunct to the standard therapy and potentially avoided the need of a colectomy.

The use of intracolonic vancomycin appears to be a safe and effective therapy for the patients with CDI and is particularly useful in the cases with toxic megacolon or ileus which would prevent oral antibiotics from reaching the distal colon [22, 23]. The largest case series report involved only 9 patients and the common dosage given was 2-3 g/day with dosing intervals of 4–12 hours [23]. However, the optimal duration of administration remains unknown and it varied from 4 to 17 days from the available case description. A reasonable strategy employed in our patient is to keep the intracolonic vancomycin until clinical response [10], or when surgical intervention is necessary. Before the generation of intracolonic vancomycin usage, should a patient require colectomy for toxic megacolon or for a colon perforation, the mortality was traditionally up to 50% [24]. In recent years, there are a few publications showing effectiveness of vancomycin enema in treatment treating severe CDI and reducing the need for laparotomy. In a large case series of CDI in 1980s from US, eight patients with clinical evidence of colonic distension suggesting possible toxic megacolon were treated with vancomycin enema in combination with intravenous metronidazole and oral vancomycin; this approach was successful in six out of the eight patients and the response period ranges from 4 to 17 days [8]. A report in 2000 found a complete or partial success rate of 57% to 71% in eight patients with fulminant pseudomembranous colitis with ileus and toxic megacolon by administrating intracolonic vancomycin in addition to decompressive colonoscopy [25]. Another case series which studied nine patients with severe* C. difficile* colitis reviewed complete disease resolution without any recurrence, need for surgical intervention, or* C. difficile* related death in eight patients after adjunctive intracolonic vancomycin [23]. These figures illustrate an increased overall survival and reduced need for surgical intervention compared to the historical data. This suggests an aggressive and a prompt medical treatment with intracolonic vancomycin in patients with severe CDI could largely improve the survival rate, prevent surgery, and serve as an alternative if surgery carries a high risk.

In conclusion, we report a case of fulminant CDI in a cirrhotic patient with an atypical endoscopic appearance, with multiple polypoid lesions causing colonic stricture which dramatically responded to adjuvant intracolonic vancomycin. This case coincides with the available literature discussing CDI as more virulent in cirrhotic patients and rectal vancomycin as an effective means of therapy in severe diseases. The polypoid and stricture appearance is probably representative of an extreme spectrum of severe colitis with massive mucosa inflammation and underlying tissue swelling, so that other clinical signs of fulminant disease must be sought and managed accordingly in such case. Although the intracolonic vancomycin was not given in this case at the first place, the addition of this agent was likely to induce the clinical response and salvage the patient from colectomy due to progressive colitis and sepsis. However, more experience and larger controlled trials are warranted to establish the optimal dosage, duration, and roles for the use of this adjunctive therapy.
